# Knowledge and Skill Retention of In-Service versus Preservice Nursing Professionals following an Informal Training Program in Pediatric Cardiopulmonary Resuscitation: A Repeated-Measures Quasiexperimental Study

**DOI:** 10.1155/2013/403415

**Published:** 2013-07-22

**Authors:** Jhuma Sankar, Nandini Vijayakanthi, M. Jeeva Sankar, Nandkishore Dubey

**Affiliations:** ^1^Department of Pediatrics, PGIMER, Dr. Ram Manohar Lohia Hospital, New Delhi 110001, India; ^2^Department of Pediatrics, All India Institute of Medical Sciences, New Delhi 110029, India

## Abstract

Our objective was to compare the impact of a training program in pediatric cardiopulmonary resuscitation (CPR) on the knowledge and skills of in-service and preservice nurses at prespecified time points. This repeated-measures quasiexperimental study was conducted in the pediatric emergency and ICU of a tertiary care teaching hospital between January and March 2011. We assessed the baseline knowledge and skills of nursing staff (in-service nurses) and final year undergraduate nursing students (preservice nurses) using a validated questionnaire and a skill checklist, respectively. The participants were then trained on pediatric CPR using standard guidelines. The knowledge and skills were reassessed immediately after training and at 6 weeks after training. A total of 74 participants—28 in-service and 46 preservice professionals—were enrolled. At initial assessment, in-service nurses were found to have insignificant higher mean knowledge scores (6.6 versus 5.8, *P* = 0.08) while the preservice nurses had significantly higher skill scores (6.5 versus 3.2, *P* < 0.001). Immediately after training, the scores improved in both groups. At 6 weeks however, we observed a nonuniform decline in performance in both groups—in-service nurses performing better in knowledge test (10.5 versus 9.1, *P* = 0.01) and the preservice nurses performing better in skill test (9.8 versus 7.4, *P* < 0.001). Thus, knowledge and skills of in-service and preservice nurses in pediatric CPR improved with training. In comparison to preservice nurses, the in-service nurses seemed to retain knowledge better with time than skills.

## 1. Introduction

Of all healthcare professionals, nurses are often the first to discover a patient of cardiopulmonary arrest (CPA) in any part of the hospital, be it the “emergency” or the “in-patient” wards. Therefore, it is needless to say that their competency in cardiopulmonary resuscitation (CPR) is a critical factor in determining successful outcomes in patients who develop CPA [1–3]. Cardiopulmonary resuscitation (CPR) is a procedure to support and maintain breathing and circulation for an infant, child, or adolescent who has stopped breathing (respiratory arrest) and/or whose heart has stopped (cardiac arrest) [4, 5]. Of all patient care areas, the emergency and ICU are the places where most of the CPAs are witnessed (95%−98% of CPAs occur in these areas in our institute), as the critically ill or injured children are admitted or transferred to these areas. Therefore, the competence of the nurses posted in these acute care areas becomes very important. Competency in CPR is defined as *acquisition* and *retention *of CPR cognitive knowledge and skills in order that health care professionals will be able to perform CPR in a CPA situation [4–6]. However, evidence is compelling to show that CPR knowledge and skills are poorly retained across nursing populations [7–10]. 

Several studies had evaluated the knowledge and CPR performance skills of either in-service or preservice nurses individually and found decline in both domains with time. Irrespective of the status of baseline training of the participant, these studies showed considerable decrease in knowledge recall and skill performance even after short periods of time following completion of the courses [6, 8, 11, 12]. For example, a study on evaluation of abilities of nurses to retain basic/advanced life support skills and theoretical knowledge found that theoretical knowledge was retained but performance skills of the nurses degraded quickly [13]. In another study from Bahrain [14], the authors observed that only 7% of participants passed the knowledge test. A systematic review of studies on CPR retention over a 9-year period revealed poor knowledge and skills retention [10]. All the studies included in the systematic review had dealt with adult CPR. Given the fundamental differences between the steps in adult and child CPR, the findings of these studies cannot be extrapolated to health professionals involved in care of sick children [15–17]. To enumerate a few differences between child and adult CPR, the compression ventilation ratio for adult CPR is universal (30 : 2) irrespective of whether there is one rescuer or 2 rescuers whereas that for infant/child CPR is 15 : 2 for 2 rescuers and 30 : 2 for single rescuer. It is much simpler to remember one ratio instead of two. Within the pediatric age group the compression depth is different for infants and for children (4 cm or 1.5 inch and 5 cm or 2 inches resp.) whereas it is 2 inches or 5 cm in adults. The compression technique is not only different for infants and children, within infancy it is different for 1 rescuer and 2 rescuers (two fingers versus 2 thumbs encircling technique). The recommendation for the site of chest compression is also different for infants and children. With so many differences in the steps of CPR it is much more challenging to remember the steps and the sequence of skills in infants/children than adults. Therefore, it may not be surprising to find that the overall performance of nurses in terms of knowledge and skills in infant/child CPR may be poorer in comparison to their counterparts who are required to perform adult life support. However, there is a dearth of knowledge in this regard, and we could identify only one study till date by West [18] which assessed the knowledge and skills of 6 registered nurses in infant/child CPR. 

All the aforementioned studies had evaluated only a particular group of nurses (e.g., Madden [19] evaluated only preservice nurses while Smith et al. [13] evaluated in-service nurses), but none have compared both groups in the same setting. Studies have compared the knowledge and skill retention of nurses with those of doctors in basic and advanced life support [20] and have found that nurses as first responders scored higher than the doctors in basic life support, while it was the other way round for advanced life support in which the doctors scored better than the nurse. The authors hypothesized that there would be differences between the nurses' and doctors' knowledge of CPR guidelines based on their motivation to learn and experience. On similar lines, we hypothesized that there would be important differences between the level of knowledge and skill retention with time, between in-service nurses and preservice nurses due to differences in their professional background and motivation to learn. We presumed that since in-service nurses are actively involved in resuscitation on a day-to-day basis their skills would be better than preservice nurses whose major academic curriculum is restricted to theoretical learning. On the other hand, student nurses would be better at retaining knowledge as they have the time and willingness to assimilate knowledge through reading the course material. They might therefore require more hands on training as their participation in infant/child CPR is most of the times only in assisting the in-service staff. We wanted to explore how these two groups would differ with regard to knowledge and skill acquisition and retention. We also wanted to decide the frequency of retraining/update so as to maintain quality of CPR performance and also a method of training suited to the needs of the particular group. 

With this background, we aimed to compare the in-service and the preservice nurses' (a) baseline knowledge and skills in infant and pediatric CPR, (b) competence in acquiring the necessary knowledge and skills following recommended teaching methods, and (c) retention of knowledge and skills acquired following training at a prespecified time point.

## 2. Methods

### 2.1. Design and Setting

A prospective, repeated-measures (before and after) quasiexperimental design was selected to test participants at 3 pre-specified time points (initial, immediately aftertraining, and at 6 weeks aftertraining). The study was conducted in the emergency and intensive care units of the department of pediatrics of a tertiary care teaching hospital between January and March 2011 after obtaining clearance from the “Institutional Ethics Committee.”

 In situations of cardiac arrest the nurses posted in these areas perceive their roles in almost all aspects of CPR except insertion of advanced airway and defibrillation. The frequency of cardiac arrest in the ICU and emergency are approximately 15–20/month and 20–30/month respectively. After performing CPR in the emergency the children are transferred to the ICU for post resuscitation care.

### 2.2. Participants

The study participants comprised of registered nurses (in-service nurses) and undergraduate nursing students (preservice nurses) posted in acute care areas such as emergency and pediatric intensive care unit of the department of pediatrics. In-service nurses in our institute are registered staff nurses posted in a particular area of the hospital during their service period. Preservice nurses are student nurses pursuing their “bachelors” course in nursing with two years of theoretical learning followed by one year of practical learning in various specialties of medicine. All of the 28 staff nurses posted in ICU and emergency participated in the study. The nursing students (46 in number) were in their final year of college and were posted in the department of pediatrics during the study period. The participation was voluntary for both groups. We obtained written informed consent from all the participants before starting the study.

### 2.3. Assessment Tools (Pre/Postintervention)

*Knowledge* was assessed initially, immediately aftertraining and again at 6 weeks aftertraining by a structured questionnaire containing 15 knowledge and practice questions (each given 1 mark for correct answer and 0 for wrong answer) validated by 2 national level pediatric advanced life support (PALS) instructors independently. The pass percentage for the study was as per the recommendation of 84% [15, 16] of the total 15 marks. Multiple-choice questions on airway, chest compressions (rate, depth, and site), order of resuscitation, drug administration, and one-rescuer and two-rescuer recommendations were used (see Appendix 1 in Supplementary Material available online at http://dx.doi.org/10.1155/2013/403415).

*Skills *were assessed at the same time points as knowledge by structured observation of CPR performance skills on infant manikin (Resusci Baby Basic Skill Guide version) by a national level PALS instructor not involved in the training phase. The skill guide unit, an electronic evaluation system placed inside the manikin, helped in evaluating correct and incorrect performances. The skill guide unit comes with the baby manikin and provides immediate and objective feedback on key CPR elements such as volume of ventilation and correct position and depth of chest compressions. An established observation checklist as given in the basic life support (BLS) guidelines for health care providers containing 12 skills in the recommended order (1 mark for correct and 0.5 for partially correct performance) was used [17]. The maximum marks in skill test were thus 12 marks. No marks were awarded for not attempting the skill and for incorrect performance (Appendix 2 in Supplementary Material). For partially correct performance the following definitions were used for each component of the check list (Appendix 3 in Supplementary Material). Only infant case simulations were provided to maintain reliability. 

### 2.4. Intervention/Training

The training program on pediatric CPR comprised of distribution of resource material 3 weeks before the initial test a 6-hour course after the initial test in the form of lectures, video demonstrations, instructor-led discussions, and case simulation as per the American Heart Association-Pediatric Advanced Life Support guidelines 2005 [15, 17]. The resource material was in the English as all the participants are proficient in English language in our hospital. The resource material was distributed after enrolling the nurses in the study, 3 weeks before the initial test and training. This was in accordance with the PALS courses conducted in our country in which the resource material is distributed 3-4 weeks in advance of the 2-day course. Demonstration of CPR performance skills (one-rescuer and 2-rescuer scenarios) using pediatric and infant manikin (batches of 6 students/session; 6 sessions per day of 60 minutes each) was carried out by the principal investigator (PI) who is also a certified PALS instructor. During each training session lasting one hour each, the PI first demonstrated the BLS skills on infant and pediatric manikin for 20 minutes to the 6 students and then asked them to practice while watching for the remaining 40 minutes (6-7 minutes per candidate). 

### 2.5. Procedures

In the initial test, the participants' knowledge was tested using a validated questionnaire. For assessment of skills each participant was provided with single-rescuer and two-rescuer case simulations. Assessment of their skills was performed in isolation of their peers. The process of evaluation of each participant took 3 minutes duration. This was followed by the training as described above (see Section 2.4). Following the training, the participant's knowledge and skills were reassessed in the same manner as in the initial test at 2 time points such as immediately aftertraining and at 6 weeks aftertraining. For ensuring greater reliability, the same scenarios, test sheet, and questionnaire were used throughout the study. The questions used were the same for all the 3 tests. However, the orders of the questions as well as the order of the multiple choices for every question were changed for every test. Neither the answers nor the scores of the written tests were revealed to any of the participants until the end of the study. However, after each evaluation the participants were given individual feedback on their overall performance with emphasis on their area of weakness.

### 2.6. Data Collection and Analysis

The demographic variables such as age of the nurses, years of experience, previous training in CPR, and number of resuscitations assisted were recorded in a proforma. Data were entered into Microsoft Excel 2007 and analyzed using Stata 11.0 (StataCorp, College Station, TX). Categorical data are presented as number (%) while continuous variables are presented as mean (SD) or median (interquartile range) based on the distribution of data. Statistical analysis was performed using Student's *t*-test for continuous variables and Chi square test for categorical variables. We also performed generalized estimating equations (GEE)—population averaged model to estimate the magnitude of change between the two groups after adjusting for baseline scores and to determine the interaction between the time points and the groups. A *P* value of 0.05 was considered significant. The participants who dropped out at any point of the study were excluded from the analysis at that time point. 

## 3. Results

### 3.1. Baseline Characteristics

**Figure 1:**
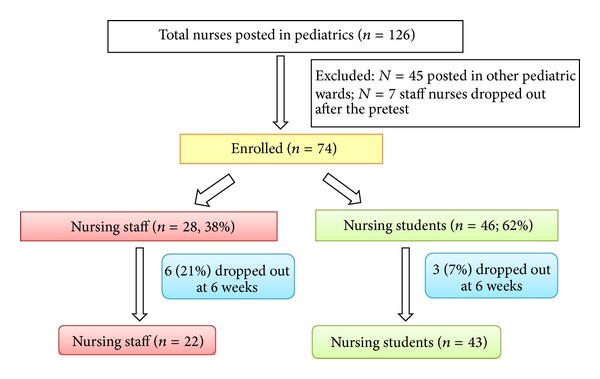
Study flowchart.

**Figure 2:**
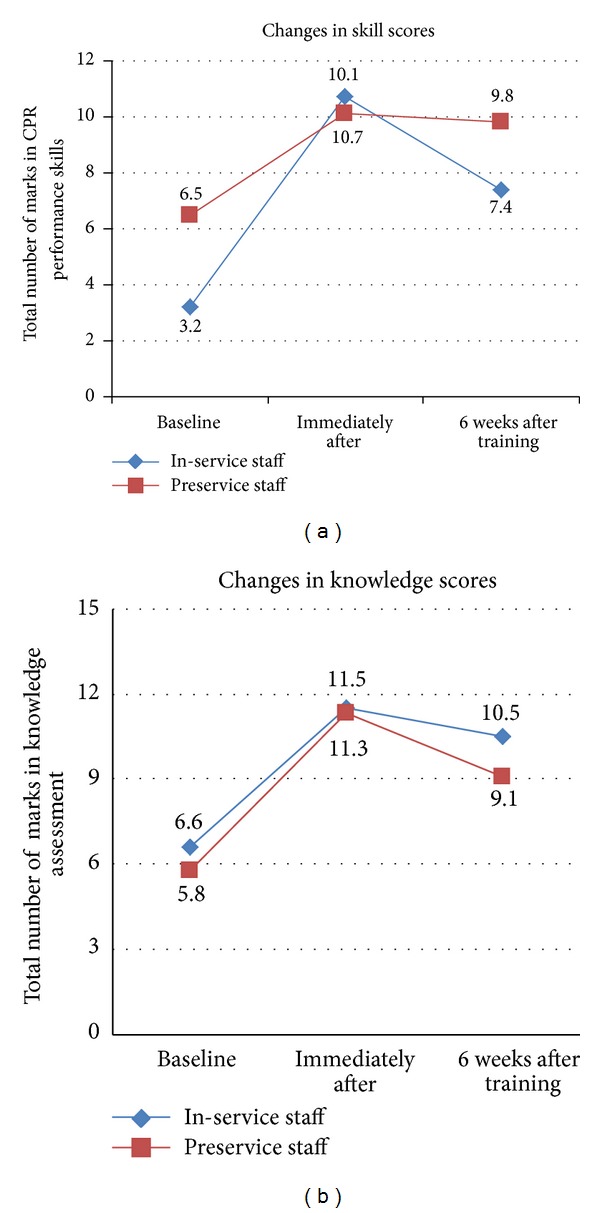
Change in knowledge and skill scores between the two groups with time.

A total of 74 nursing personnel—28 in-service and 46 preservice nurses—participated in the study. However, at 6 weeks, 6 in-service and 3 preservice nurses dropped out of the study leaving us with 22 and 43 in these two groups, respectively, for the 6 weeks aftertraining assessment (Figure 1). The 6 staff nurses who dropped out quoted personal reasons such as their children's education or leaving station as the primary reasons. The baseline characteristics of the participating nurses at the beginning of the study are described in Table 1. There were significant differences between the two groups with regard to their experience in performing CPR and level of training before the study with in-service nurses being more experienced than their counterparts. The initial knowledge and skill scores were also different between the two groups (Table 2 and Figure 2). 

### 3.2. Primary Outcomes

#### 3.2.1. Knowledge and Skills Scores Immediately Post-Training (T1)

The knowledge and skills scores improved after training in both groups. The mean knowledge scores of the in-service nurses improved from 6.6 to 11.5, while of the preservice group improved from 5.8 to 11.3; the difference being statistically significant for both groups. The skill scores also improved immediately aftertraining from 3.2 to 10.7 in the in-service group and 6.5 to 10.1 aftertraining in the preservice group; again the difference was statistically significant for both groups. There was, however, no difference in either the knowledge scores (mean difference: 0.24; 95% CI: −0.6 to 1.17) or the skill scores (0.54; 95% CI: −0.11 to 1.19) between the groups immediately aftertraining (Table 2).

#### 3.2.2. Knowledge and Skills Scores at 6 Weeks after Training (T2)

The knowledge and skill scores at 6 weeks aftertraining were significantly different between the in-service and preservice nurses. While the in-service group had higher mean knowledge scores (10.5), their mean skill scores (7.4) were found to be significantly lower than the preservice group (9.1 knowledge and 9.8 skill score). 

#### 3.2.3. Retention of Skills: Change from T1 to T2 between the Groups

Both the knowledge and skills scores showed a decline from T1 to T2. The magnitude of change, after adjusting for the initial scores, was not significantly different between the two groups (adjusted mean difference 0.06; *P* = 0.90 for knowledge and 0.91; *P* = 0.07 for skill scores) (Table 2). However, there was evidence of significant interaction between the magnitude of change of the scores from T1 to T2 and the groups (*P* value for interaction being 0.03 and <0.001 for knowledge and skills scores, resp.) (Table 2), suggesting that the degree of fall was different in the two groups. Thus, the in-service nurses seemed to retain knowledge better with time and the preservice nurses skills.

### 3.3. Secondary Outcomes

#### 3.3.1. Proportion Passing the Knowledge and Skill Tests

As with the mean knowledge scores, the proportion of in-service staff who passed the initial knowledge test was higher than the proportion in the preservice group (9% versus 0%,  *P* = 0.07). However, with training the proportion of participants passing the knowledge test (immediately aftertraining) was the same in both the groups (43% versus 43%, *P* = 0.51). At 6 weeks aftertraining, the performance of these two groups was similar to their initial performance with 23% of the staff nurses and only 5% of the students passing the knowledge test (*P* = 0.07). 

With regard to performance skills in infant CPR, although there was no difference in the proportion of participants passing the initial test, towards the end (i.e., at 6 weeks aftertraining) the students seemed to outperform the staff nurses with 37% of the students passing the skill test in comparison to only 9% of the nursing staff (*P* = 0.01). 

Only one student scored 100% marks in knowledge test in the immediate posttraining assessment. No participant could perform all the steps of CPR correctly and in the recommended order at any point of time. Irrespective of the group to which they belonged, most of them went wrong even after the training (i.e., immediate posttraining and 6 weeks posttraining numbers combined) either in “changing rescuer positions after 5 cycles” (*n* = 118, 85%), in the “depth of chest compressions” (*n* = 97, 70%), in the “order of resuscitation” (*n* = 83, 60%), or in not being able to “allow full chest recoil” after each compression (*n* = 78, 56%).

## 4. Discussion

In this repeated measures, before and after study we found that the knowledge and skills of in-service and preservice nurses posted in acute care areas seemed to improve following training in pediatric CPR. However, by 6 weeks, the knowledge and skills had started to decline although they continued to remain significantly higher than their initial values. Thus, our training program had improved the overall competency of these two groups of nurses in pediatric CPR but they failed to retain the same competency even for a short duration such as 6 weeks. Our study findings are therefore in agreement with most of the published adult studies and the only pediatric study till date in this regard [6–14, 18].

Between the two nursing subgroups, the in-service nurses seemed to fare better in the knowledge domain and the preservice nurses better at skills at the beginning as well as at the end of the study. We observed that similar to previous studies only knowledge was better retained with time [6, 8, 18, 21] in the in-service group. This was in contrast to our hypothesis, according to which we expected that the in-service nurses would be better in both domains as compared to the preservice nurses at any point of time, given the differences in their professional background. One of the probable and possibly the major reason for this finding could be that the in-service nurses might have learnt incorrect skills during their nursing curriculum or service period, and it is much more difficult to change learned behavior than to learn completely new behavior or skills. Another reason could be probably that the method of training was not suited to the needs of the participants of the study particularly the in-service nurses. This was despite the use of standard teaching methods as per PALS recommendations [15–17] used in the provider courses. This was reflected in the reason given by most of the staff nurses for scoring such poor marks, which was lack of time to read the material at home as they had to attend to family chores, rather than devote time to read the material. In fact, only 2 of the staff nurses had read the resource material before taking the initial test. Probably, a method in which the nurses were made to go through the entire course material in the classroom itself with debriefing sessions by each one of them would have yielded better results. Finally, the complexity of steps involved in infant and child CPR (see Section 1) in comparison to adult CPR steps might have played an important role in the whole process. Also, one must understand that this was the first time that most of the study participants had undergone such training and test. Therefore, to expect them to master the skills of pediatric CPR after a single training session would be unreasonable. 

In stark contrast to the in-service nurses, we observed that despite not being actively involved in performing CPR, the preservice nurses—by their sheer self-motivation and willingness to learn—retained the skills learnt during the training session better than the staff nurses. Learning the steps for the first time and in the correct way probably helped them to succeed. Although their mean knowledge scores were lower than those of the in-service nurses, the difference may not be clinically significant (mean score was only 1.4 points lower than that of the in-service nurses). Thus it *seems* that overall the preservice nurses fared better than the staff nurses in retaining both knowledge and skills of pediatric CPR with time. 

Ours is the largest study that had evaluated the knowledge and skills of pediatric nursing personnel posted in acute care areas in infant/child CPR and the first study to compare the same between in-service and preservice nurses. The study, however, has several limitations; firstly, nearly 20% of the staff nurses as compared to 8% of the student nurses dropped out of the study which probably affected the mean score of the respective groups at 6 weeks aftertraining. Secondly, we did not have a control group, and therefore the baseline knowledge and skills of the participants could have influenced the posttraining assessment results. Moreover, although we took adequate measures to ensure that bias due to use of the same questionnaire in all the tests was minimized, we cannot for sure rule out a potential bias due to candidates remembering the questions in the 6 weeks posttraining assessment. Perhaps our biggest limitation was the method of training which was probably not suited to the needs of the in-service nurses who were less motivated to read the learning material outside their working hours. Also, although the differences in means were statistically significant they may not be large enough to be of “practical significance”, and therefore there may not have been any actual differences between the two groups. However, the larger message from this study is that there are differences between in-service and preservice nurses' knowledge and CPR skill retention and several factors may be responsible for these observed differences, the most important of these being learning the skills in the correct way the first time and self-motivation to read, learn, and perform correctly.

Based on the findings of this study we have made a departmental policy of updating all the nursing professionals posted in critical care areas every 2 months in infant/child CPR. In order to sustain the updates on a regular basis, the training is included in their day-to-day teaching curriculum and does not require them to spend additional time outside their working hours.

## Supplementary Material

Appendix 1 is the questionnaire used for the study purpose. Appendix 2 is the skill check list. Appendix 3 is the definitions for partially correct skills.

## Figures and Tables

**Table 1:** Baseline characteristics of participating nurses.

Variables	In-service nurses (*n* = 28, 38%)	Preservice nurses (*n* = 46, 62%)	*P* value
Mean (SD) age in years	30 (7)	22 (2)	<0.001
Years of experience in pediatrics		0	<0.001
<1 year	22 (62)		
1–3 years	4 (12)		
>3 years	2 (25)		
Prior training in CPR	7 (20)	1 (2.2%)	0.01
No of resuscitations assisted in last 6 months		0	<0.001
>10	18 (51)		
<10	17 (48)		
Prior experience in the following steps of resuscitation*		0	<0.001
Airway	15 (42.8)		
Chest compression	15 (42.8)		
Drug administration	20 (57)		
Defibrillation	1 (2.8)		

Data is expressed as number (%) unless specified otherwise; SD: standard deviation; *not mutually exclusive.

**Table 2:** Knowledge and skills scores of the two groups at different time points.

Variable	In-service nurses mean (SD)	Preservice students mean (SD)	Mean difference (95% CI); *P* value	Change from T1 to T2 *between* the groups; adj. mean difference* (95% CI); *P* value
(1) Knowledge scores				
Initial (T0) Median (range)	6.6 (2.6)^1i^	5.8 (1.5)^1p^	0.86 (−0.1 to 1.8); **0.08**	—
Immediately after training (T1) Median (range)	11.5 (1.9)^2i^	11.3 (2)^2p^	0.24 (−0.6 to 1.17); 0.60	0.06 (−0.87 to 0.98); 0.90
6 weeks after training (T2) Median (range)	10.5 (2.4)^3i^	9.1 (2)^3p^	1.44 (0.33 to 2.54); **0.01**	
Interaction between change in scores from T1 to T2 and designation	*P* = 0.03			

(2) Skill scores				
Initial (T0) Median (range)	3.2 (2.3)^1i^	6.5 (1.6)^1p^	−3.27 (−4.3 to −2.23); **<0.001**	—
Immediately after training (T1) Median (range)	10.7 (1.3)^2i^	10.1 (1.1)^2p^	0.54 (−0.11 to 1.19); 0.10	0.91 (−0.08 to 1.91); *0.07 *
6 weeks after training (T2) Median (range)	7.4 (2.6)^3i^	9.8 (1.6)^3p^	−2.4 (−3.43 to −1.34); **<0.001**	
Interaction between change in scores from T1 to T2 and designation	*P* < 0.001			

SD: standard deviation; CI: confidence interval.

*Adjusted for baseline (T0) values using generalized estimating equation (GEE)—population averaged model.

1i-, 2i-, and 3i-proportion of in-service nurses evaluated at T0, T1, and T2 were 28, 28, and 22, respectively.

1p-, 2p-, and 3p-proportion of preservice nurses evaluated at T0, T1, and T2 were 46, 46, and 43, respectively.
