# Supplementary material for: Knowledge and Skill Retention of In-Service versus Preservice Nursing Professionals following an Informal Training Program in Pediatric Cardiopulmonary Resuscitation: A Repeated-Measures Quasiexperimental Study
Source: Biomed Res Int. 2013 Jul 22;2013:403415. doi: 10.1155/2013/403415 (PMC3736513; doi:10.1155/2013/403415)
Supplement: Supplementary file 1 — Appendix 1 is the questionnaire used for the study purpose. Appendix 2 is the skill check list. Appendix 3 is the definitions for partially correct skills. [file 403415.f1.doc]

**Knowledge and Skill Retention of In-service vs. Pre-service Nursing Professionals Following an Informal Training Program in Pediatric Cardiopulmonary Resuscitation: A Repeated- measures Quasi-experimental study**

**Appendix 1. Questionnaire used for the study purpose**

| 1. **Questions** |
| --- |
| 1. Good quality CPR includes the following EXCEPT-a) push hard, push fast (100/min); b) allow full chest recoil after each compression; c) minimize interruptions; d) hyperventilate 2. The depth of chest compressions in children >8 years of age should be- a) ½ to 1/3 of the depth of the chest; b) 1½ to 2 inches c) 1/3 of the depth of the chest 3. A 3 year old child was brought to the emergency department with no spontaneous breathing. Junior resident has done the initial steps of opening airway but the child is still breathless. What is the next step?- a) chest compressions; b) endotracheal intubation; c) bag and mask ventilation 4. The site of chest compression in infants is- a) Centre of the chest between the nipple; 5. b) One finger breath below the nipple line; c) Just below the nipple line 6. Rate of compression to ventilation in case of single rescuer CPR in a 5 year old child is- a) 5:1; b) 15:2 c) 30:2 7. For how long the chest compression should continue in child/infant 1 rescuer CPR before next assessment- a) 3 cycles or 1 mm; b) 3 cycles or 2 min; c) 5 cycles or 1 min; d) 5 cycles or 2 min 8. The depth of chest compressions in children < 1 year of age should be - a) ½ to 1/3 of the depth of the chest; b) 1½ to 2 inches ; c) 1/3 of the depth of the chest 9. The depth of chest compressions in children 1 – 8 years of age should be- a) ½ to 1/3 of the depth of the chest; b) 1½ to 2 inches and c) 1/3 of the depth of the chest 10. Chest compression in an infant by lone rescuer is done by- a) heel of one hand 11. b) heel of one hand, other hand on top ; c) two figures; d) two thumbs 12. Brain withstands hypoxia for – a) 5 mins; b) 5 secs ; c) 10 secs ; d) 10 mins 13. The order of assessing airway patency? – a) look, feel, listen b) look, listen, feel c) 14. feel, listen, look 15. To check for cardiac arrest in an infant, which artery is palpated for pulse check?- a) radial b) femoral d) carotid d) brachial 16. While checking for breathing during CPR of the look, listen & feel, what does 'look' means- a) oral secretions; b) nasal flaring; c) chest retractions d) chest rise & fall 17. Name the maneuver for removal of Foreign Body in a responsive infant- a) Heimlich maneuver b) back slaps and chest thrusts c) CPR d) blind finger sweep 18. In-CPR, interruptions in chest compression should be minimal and not more than?- a) 19. 10 seconds; b) 5 seconds; c) 13 seconds; d) 20 seconds |

**Appendix 2**. CPR checklist and method of scoring

| **Steps of CPR** | **Skill component** | **Value** | **Points** |
| --- | --- | --- | --- |
| **Step 1** | Checks for response and calls for help | Correct  partially correct  Incorrect/ not performed (NP) | 1  0.5  0 |
| **Step 2** | Opens airway by head tilt chin lift | Correct  partially correct  Incorrect /NP | 1  0.5  0 |
| **Step 3** | Checks if breathing or not (time max-10 seconds) | Correct  partially correct  Incorrect /NP | 1  0.5  0 |
| **Step 4** | Gives two effective breaths looking for chest rise( sealing around the mouth proper or not) | Correct  Incorrect / NP | 1  0 |
| **Step 5** | Checks for brachial pulse in infant( correct method or not-max time 10 secs) | Correct  partially correct  Incorrect /NP | 1  0.5  0 |
| **Step 6** | Locates position for chest compressions properly | Correct  Incorrect/NP | 1  0 |
| **Step 7** | Delivers one cycle correctly( 30 compressions in ~23 secs)[depth, rate, release all three should be correct] | Correct  Incorrect /NP | 1  0 |
| **Step 8** | Gives 2 breaths and continues | Correct  Incorrect /NP | 1  0.5  0 |
| **Two rescuer scenario provided (PI acts as second rescuer)** | | | |
| **Step 9** | 1^st^ rescuer delivers 15 chest compressions using two thumb technique | Correct  Incorrect/NP | 1  0 |
| **Step 10** | 1^st^ rescuer waits till second rescuer gives two breaths | Correct  Incorrect /NP | 1  0 |
| **Step 11** | After 5 cycles watch if 1^st^ rescuer switches places or not | Correct  Incorrect /NP | 1  0 |
| **Step 12** | Watch if the first rescuer asks for AED or not | Correct  Incorrect /NP | 1  0 |

**Appendix 3.** Definitions for partially correct performance of few of the skills

| **Skill** | **Definition of partially correct performance** |
| --- | --- |
| **Checks for response and calls for help** | checks for response and calls for help but not in the correct order |
| **Opens airway by head tilt chin lift** | Performs head tilt and chin lift but either hyperextends the neck or lifts chin using the mandible |
| **Checks if breathing or not** | Does not perform look listen and feel in the correct order although performs each component correctly |
| **Checks for brachial pulse in infant** | Checks pulse correctly but in <5 seconds |
